# The Use of Porous Scaffold as a Tumor Model

**DOI:** 10.1155/2013/396056

**Published:** 2013-09-11

**Authors:** Mei Zhang, Philip Boughton, Barbara Rose, C. Soon Lee, Angela M. Hong

**Affiliations:** ^1^Department of Radiation Oncology, Royal Prince Alfred Hospital, Sydney, NSW 2050, Australia; ^2^Sydney Medical School, The University of Sydney, Sydney, NSW 2006, Australia; ^3^The Institute of Biomedical Engineering and Technology, The University of Sydney, Sydney, NSW 2006, Australia; ^4^Department of Infectious Diseases and Immunology, Central Clinical School, The University of Sydney, Sydney, NSW 2006, Australia; ^5^Discipline of Pathology, School of Medicine, University of Western Sydney, Richmond, NSW 2751, Australia; ^6^Cancer Pathology, Bosch Institute, The University of Sydney, Sydney, NSW 2006, Australia

## Abstract

*Background*. Human cancer is a three-dimensional (3D) structure consisting of neighboring cells, extracellular matrix, and blood vessels. It is therefore critical to mimic the cancer cells and their surrounding environment during *in vitro* study. Our aim was to establish a 3D cancer model using a synthetic composite scaffold. *Methods*. High-density low-volume seeding was used to promote attachment of a non-small-cell lung cancer cell line (NCI-H460) to scaffolds. Growth patterns in 3D culture were compared with those of monolayers. Immunohistochemistry was conducted to compare the expression of Ki67, CD44, and carbonic anhydrase IX. *Results*. NCI-H460 readily attached to the scaffold without surface pretreatment at a rate of 35% from a load of 1.5 × 10^6^ cells. Most cells grew vertically to form clumps along the surface of the scaffold, and cell morphology resembled tissue origin; 2D cultures exhibited characteristics of adherent epithelial cancer cell lines. Expression patterns of Ki67, CD44, and CA IX varied markedly between 3D and monolayer cultures. *Conclusions*. The behavior of cancer cells in our 3D model is similar to tumor growth *in vivo*. This model will provide the basis for future study using 3D cancer culture.

## 1. Background

A growing number of studies recognize the limitations of two-dimensional (2D) monolayer cultures for *in vitro* cancer biology study [1–6]. The adhesion and organization of cancer cells growing in conventional 2D culture may differ from those *in vivo* in terms of proliferation, cellular signal transduction, and response to drugs and radiation [7–13]. *In vivo* cancer-associated stroma is a three-dimensional (3D) structure consisting of neighboring cells, extracellular matrix (ECM), blood vessels, immune cells, and cytokines. Recent studies of 3D culture have demonstrated that cancer cells interact extensively with their microenvironment during proliferation, angiogenesis, metastasis, and chemo- or radiotherapy [14–22]. It is therefore critical to mimic cancer cells and their surrounding environment during *in vitro* study. 

Several strategies have been used to develop an *in vitro* cancer model that can accurately represent *in vivo* conditions. The most commonly used matrix gel 3D culture method involves the encapsulation of cancer cells within collagen, laminin-based matrix, or hydrogel. Using matrix gel in multiwell or transwell plates, breast cancer and ovarian cancer cell lines have been studied for gene expression, cellular signal pathways, angiogenesis, and chemotherapy response [22–25]. The semisolid matrix gel also provides a very useful tool to study tumor metastasis [18]. Recently there has been great interest in using multicellular tumor spheroid models to study gene expression and antitumor treatment therapy [14, 20, 23, 26, 27]. Spheroid culture was developed 40 years ago and gradually more advanced models have been created to accommodate the fast-growing field of cancer targeted therapy. 

In contrast to the matrix and spheroid technologies, 3D models using porous scaffolds are relatively new. In 2007 Fischbach et al. established a 3D model using oral squamous cell carcinoma and compared the 3D model both *in vitro* and *in vivo* conditions with very encouraging results [15]. Since then porous collagen scaffolds, silk porous scaffolds, and nanoparticle-incorporated scaffolds have been developed to mimic angiogenesis, tumorigenicity, cancer metastasis, cancer stem cell properties, and drug uptake and release [28–39]. However, only a few solid tumors have been tested using these approaches [15, 40]. There is a vast demand for 3D scaffold models for solid tumors directed at improved cellular attachment for cancer biology study.

The aim of the present study was to establish a 3D cancer model using a synthetic composite scaffold (Variotis Tissue Scaffolds, Biometic, Sydney, Australia). This scaffold comprises highly interconnected and porous polyester-based material with a pore size in excess of 100 *μ*m. It was initially developed for cartilage repair and has been used in chronic wound-healing studies. Here we studied the growth pattern of a non-small-cell lung cancer cell line using this scaffold compared with standard monolayer culture.

## 2. Materials and Methods

### 2.1. Cancer Cells

The NCI-H460 cell line (a human non-small-cell lung cancer cell derived from metastatic pleural fluid) was obtained from American Type Culture Collection (Manassas, USA). NCI-H460 has been studied extensively in 2D culture [41, 42]. This cell line was chosen because of its metastatic potential [41, 43, 44]. 

### 2.2. Standard Monolayer Culture

NCI-H460 cells were cultured in RPMI1640 (GIBCO, Life Technologies, Victoria, Australia) supplemented with 2 mM glutamine and 5% Fetal Calf Serum (FCS, Interpath Services, Melbourne, Australia) at 37°C in 5% CO_2_/humidified air. All chemicals were sourced from Sigma (Sigma-Aldrich, Sydney, Australia).

### 2.3. 3D Scaffold Culture

Variotis Tissue Scaffolds (Biometic, Sydney, Australia) were cut into 100 mm^3^, sterilized with UV radiation for 60 minutes, and then placed in 2 ml eppendorf tubes. A modified high-density random seeding method was used [45–47]. Briefly NCI-H460 cells were grown in T75 flasks; when the culture reached 80% confluence, cells were trypsinized, washed with phosphate buffered saline (PBS), and counted. Cell suspensions of 1 × 10^6^ to 3 × 10^7^ per mL were prepared in culture medium (10% FCS in Advanced RPMI1640) and 100 *μ*L of the suspension was dispended onto the scaffold slowly to ensure the cells were captured by the scaffold. The seeded scaffolds were incubated for 1 to 2 hours at 37°C without the addition of culture medium. The scaffolds were then transferred into 6-well plates (Corning Incorporated Life Sciences, Lowell, USA) with 5 mL of medium and incubated for up to 2 weeks. The medium was changed every 2 to 3 days by transferring the scaffold culture carefully to a new plate with fresh culture medium containing 20% conditioned medium. At least three scaffolds were established for each testing condition, and each experiment was performed in duplicate. To examine the presence and distribution of NCI-H460 cells on scaffolds, 1- and 3-day cultures were washed twice with PBS. The scaffolds were then fixed and stained in 0.3% methylene blue/50% ethanol solution for 30 minutes then rinsed in water. Images were taken using a Canon EOS digital camera. Data were expressed as ± standard error of mean (SEM). Statistical analyses were performed by Student's *t* test.

### 2.4. Characterization of 3D NCI-H460 Cultures

Morphological features were examined by light and fluorescent microscopy. At 1, 3, and 7 days the scaffold cultures were gently washed and fixed, stained with Hoechst 33528 for 15 minutes, and washed with PBS. Images were captured using an OLYMPUS IX51 inverted microscope. The 7- and 14-day scaffold cultures were fixed in formalin acidic acid for 3 days, embedded in paraffin, cut into 5 *μ*m sections, and stained with hematoxylin and eosin (H&E). The cells grown in monolayer were collected, made into cell blocks, and processed as for the 3D scaffolds. 

In addition, the attachment of cells on scaffold was monitored daily and quantified by cell counting after 1 and 3 days incubation, respectively. The cells were dissociated from the scaffold with 200 *μ*L of 0.25% trypsin, washed in PBS, and resuspended in 0.5 mL PBS. The numbers were counted using a hemocytometer. The cell proliferation rate was determined by a MTT (3-4,5-dimethylthiazol-2-yl)-2,5-diphenyltetrazolium bromide) assay at 1, 3, and 7 days. Briefly, the scaffold cultures were transferred into 96-well plates and incubated with 200 *μ*L solution of MTT at 1 mg/mL for 4 hours at 37°C. Then the scaffolds were transferred into 5 mL tubes with 2 to 3 mL Dimehyl sulfoxide (DMSO) to completely dissolve the formazan product converted by living cells. Eight aliquots of 150 *μ*L dissolved MTT/DMSO solution were pipetted to 96-well plates and the absorbance was read at 540 nm in a MULTISKAN EX plate reader (Thermo, Melbourne, Australia). Monolayer cultures of 5 × 10^3^ to 1 × 10^6^ cells were also seeded in 24-well plates to create a standard curve using MTT assay. The absorbance from 3D scaffold culture was then plotted on the standard curve to obtain the cell number in 3D culture. Three independent experiments were performed.

### 2.5. Analysis of Protein Expression by Immunohistochemistry

Immunohistochemistry was performed using an autostainer (Dako, Melbourne, Australia) following the manufacturer's procedures. Following treatment with antigen retrieval buffer pH6, sections were incubated for 60 minutes with the primary antibodies: Ki67 (1 : 200 Rabbit monoclonal, ab16667, Abcam, Cambridge, UK), CD44 (1 : 500 Mouse mAb, 156-3C11, Cell Signaling, Danvers, USA), or Carbonic Anhydrase IX (CA IX 1 : 1000 Rabbit anti-Human NB100-417, NOVUS, Littleton, USA). The Envision Dual Link System Peroxidase (Dako) was used as secondary antibody, and sections were counter-stained with Mayer's hematoxylin. For negative controls the primary antibodies were replaced with buffer. Staining was visualized with an Olympus Provis microscope and scored by the study pathologist (CSL).

## 3. Results

### 3.1. Initial Attachment, Distribution, and Unique Growth Patterns of NCI-H460

#### 3.1.1. Cell Number Counting

**Figure 1:**
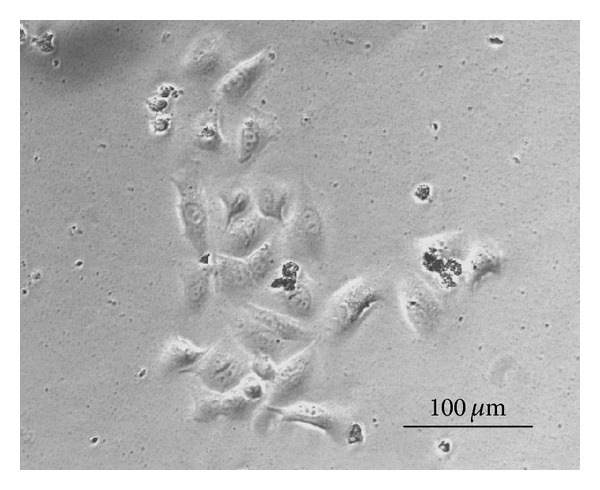
Microscopic image of the monolayer culture of NCI-H460 cells in 5% RPMI1640 after 24 hours incubation in 6-well plates.

**Figure 2:**
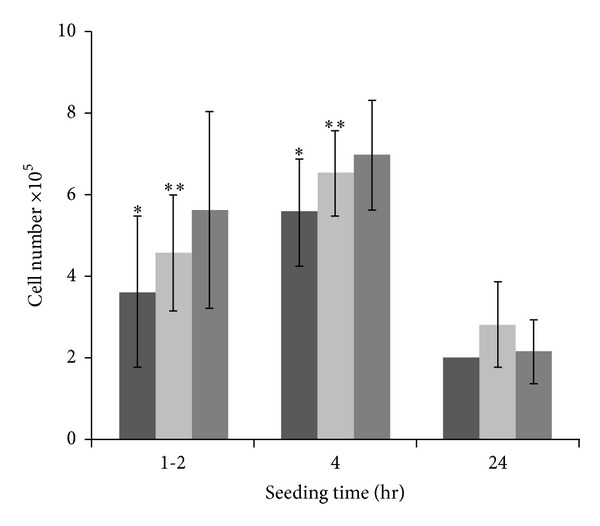
NCI-H460 cell number counted at day 4 of scaffold culture with 3 × 10^5^ (dark grey), 7 × 10^5^ (light grey) and 1.5 × 10^6^ (grey) as initial seeding cell number for 1-2 hours, 4 hours and 24 hours. There is a significant difference between 1-2 hours and 4 hours seeding; *P < 0.05, **P < 0.01.

In 2D culture, the NCI-H460 exhibited characteristics of an adherent epithelial cancer cell line; the cells were large and predominantly polygonal with a relatively high nuclear/cytoplasmic ratio that was estimated to be 0.5 to 1 (Figure 1). In 3D culture, using high-density low-volume seeding, NCI-H460 readily attached onto the scaffold without surface pretreatment. Using quantitative cell counting, we found that the highest attachment rate was 35% with 1.5 × 10^6^ cells seeded for 2 hours.  Figure 2 demonstrates that increasing the initial seeding period improved cell attachment in a time and cell density dependent manner. This was reflected in increased cell numbers at day 4. However, increasing the initial seeding time to 24 hours did not enhance cell attachment but caused cell damage possibly due to the very low volume of culture medium. 

#### 3.1.2. Methylene Blue Staining

**Figure 3:**
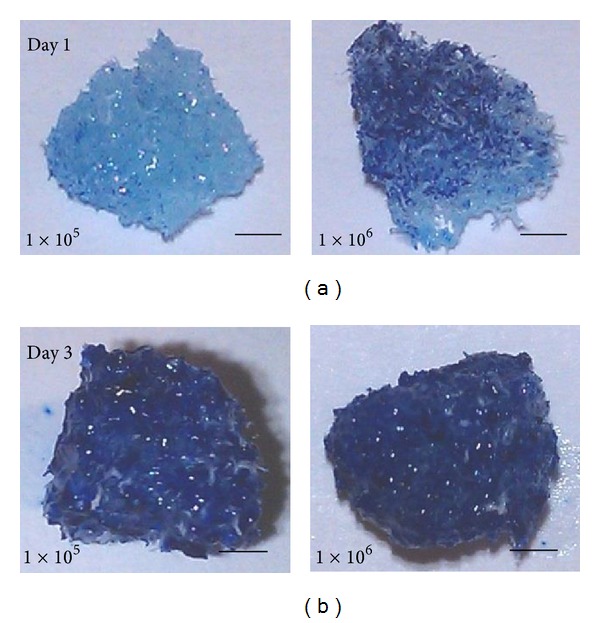
The photo images of scaffold cultures shown above are stained with methylene blue on day 1 (left panel) and day 3 (right panel) the two panels of each day show the scaffold culture seeded with 1 × 10^5^ and 1 × 10^6^ cells, respectively. Scale bars are 1 mm.

The distribution and presence of the cells on scaffolds can be seen clearly with methylene blue staining (Figure 3). On day 1, there were only a few stained cells on the surface of the scaffold seeded with 1 × 10^5^ cells; in comparison, more dark-blue staining was observed spreading randomly on the scaffold seeded with 1 × 10^6^. We noted that after incubating for 3 days the dark-blue staining covered the surface of the scaffold as well as infiltrated deep into the centre for both 1 × 10^5^ and 1 × 10^6^ scaffolds. 

#### 3.1.3. Light Microscopy

**Figure 4:**
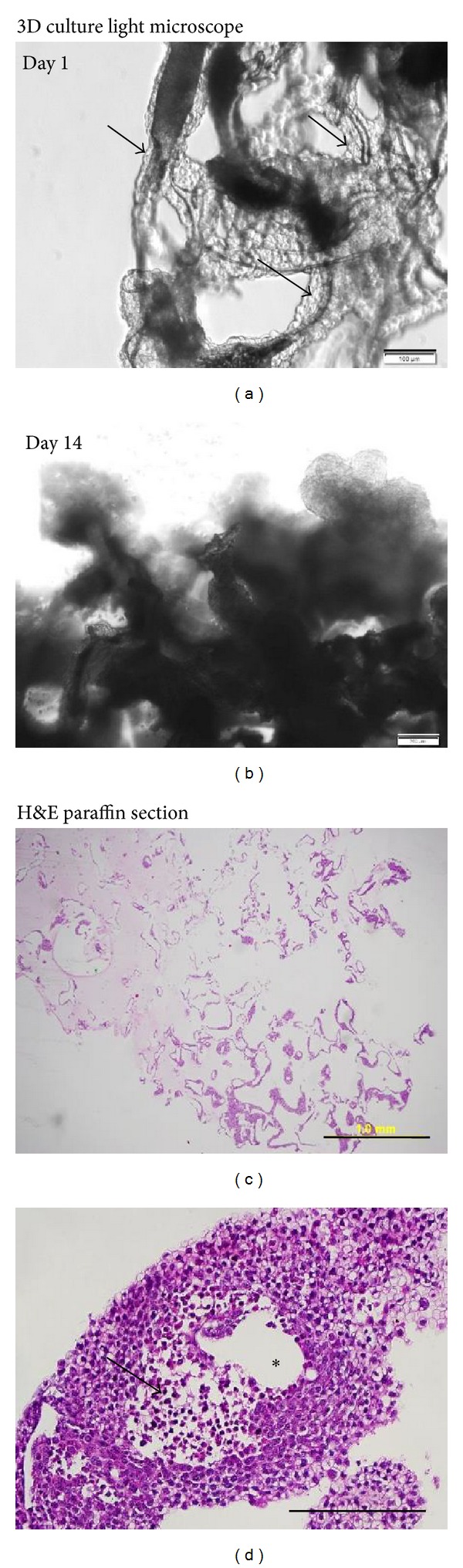
The top row shows light microscope images of NCI-H460 3D culture. (a) (Day 1) demonstrates cells (shown by arrows) that surround the scaffold (dark-shaded areas). This can be compared to (b), which shows that at day 14 multilayers of cells cover the scaffold. The bottom row shows images of H&E staining on paraffin sections of 3D culture after 1 week in culture. (c) shows the complete cross section of a scaffold with most of the large clumps located peripherally (magnification 4x). (d) is an example of a big cluster more than 500 *μ*m in diameter, including a nonviable cell centre region (shown by arrow). The clear region that is marked with an asterisk (∗) shows the location of the scaffold that dissolved in xylene (magnification 20x). Scale bars are (a) 100 *μ*m, (b) 200 *μ*m, (c) 1 mm, and (d) 200 *μ*m.

Examination of the scaffold cultures under light microscopy after overnight incubation (Figure 4(a)) showed that 20% of the scaffold surface was covered with cells. Growth extended horizontally and reached 50% surface coverage at 2 weeks culture (Figure 4(b)). H&E staining of a cross section of the scaffold culture revealed that most of the larger clumps were located towards the exterior of the scaffold (Figure 4(c)). Cancer cells growing from single to multiple layers could be seen on most fields; there were also nonviable cell populations especially in the centre of the large clumps (Figure 4(d)). 

**Figure 5:**
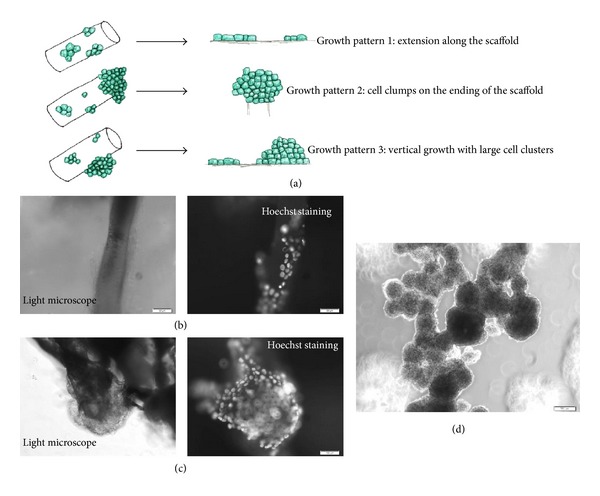
(a) Illustration of three types of growth pattern on the scaffolds. (b) to (c) showing the identical microscopic view under light (left panel) and fluorescent filters (right panel); (b) an example of form A growth; (c) an example of form (b) and (c) vertical growth; (d) large cell clumps detached from scaffold grow as tumour spheroid. Scale bars are (b) and (c) = 50 *μ*m, (d) = 200 *μ*m.

Three types of growth patterns were observed. Besides expanding horizontally, the majority of cells grew vertically to form clumps along the surface or on the end of the scaffold fiber (Figures 5(a)–5(c)). The size of the cell clusters was variable and irregular with the largest clumps reaching diameters of 500–600 *μ*m (Figure 4(b)). As the cell clumps continued to develop, they frequently separated from the scaffold culture to form structures similar to cancer spheroids (Figure 5(d)). Often the detached clumps settled onto the bottom of the culture plate and continued to grow as a monolayer. 

### 3.2. MTT Assay to Measure the Proliferation of NCI-H460 Cells on Scaffold

**Figure 6:**
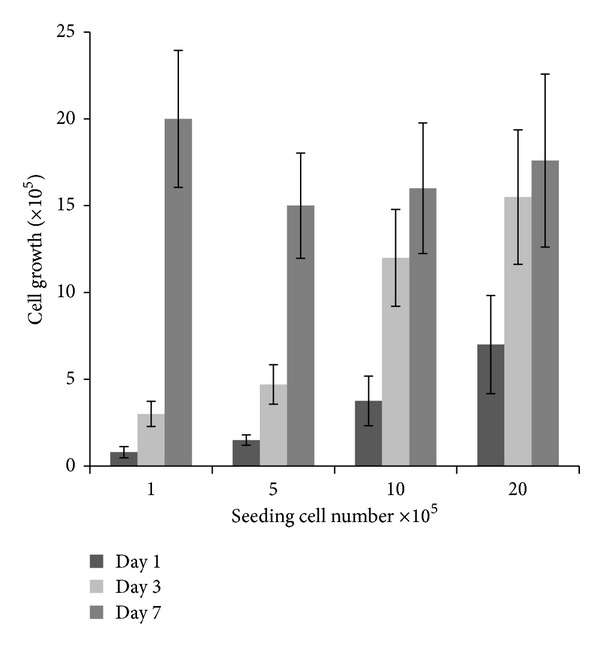
Cell proliferation measured at day 1 (dark grey), day 3 (light grey), and day 7 (grey) by MTT assay with different seeding numbers for 1-2 hours initial seeding time. The data are generated on three individual experiments with triplicate, normalised with standard curve.

As demonstrated in Figure 6, NCI-H460 cells had a slower proliferation rate in 3D scaffold than in monolayer culture; the cell doubling time in 3D culture was 2 to 3 days compared with less than 24 hours in monolayer. MTT assays confirmed 30–40% initial attachment after overnight incubation for all seeding concentrations which is indicated in the dark grey bar in Figure 6. All of the 3D culture was grown continuously in a seeding-number dependent manner. When seeded with at lower density (1 × 10^5^), the 3D culture expanded rapidly and reached 2 × 10^6^ cells on day 7 which exceeded cultures with higher initial seeding cell numbers. Similar growth behavior was observed for the 3D culture seeded with 5 × 10^5^ cells. In comparison, cells only doubled in number by day 3 for scaffolds with higher seeding numbers (1 × 10^6^ and 2 × 10^6^); after 3 days of incubation the 3D culture reached a plateau and the difference between 3 days and 7 days of incubation did not appear to be statistically significant.

### 3.3. Tumor Markers by Immunohistochemistry

**Figure 7:**
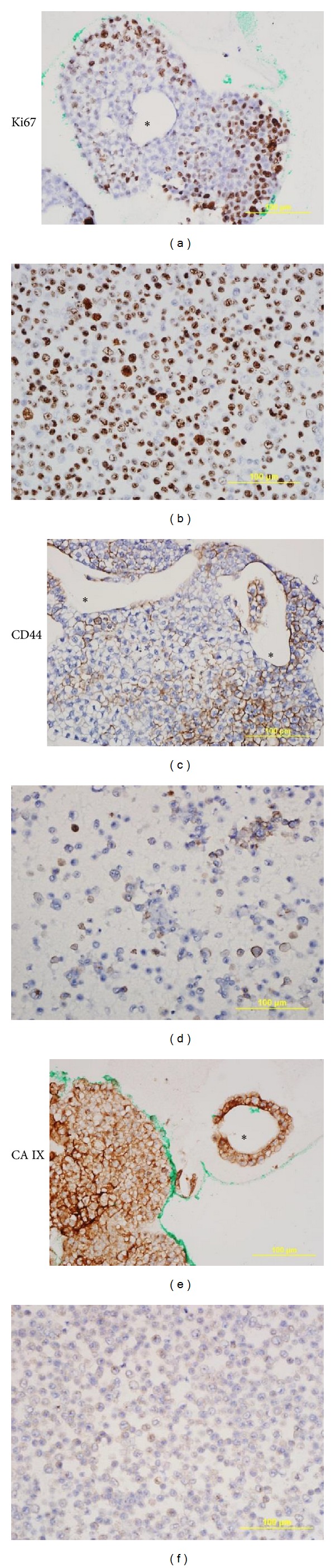
The images are representative of immunohistochemistry staining of nuclear staining of proliferation marker Ki67, membrane protein CD 44, and CA IX. The images of scaffold culture (left panel) are compared with cell pellet paraffin section (right panel). The scaffold dissolved in xylene presented as a clear region marked ∗. All scale bars are 100 *μ*m, magnification 40x.

Immunohistochemistry studies were performed on both 3D culture and monolayer cell pellets (Figure 7). For Ki67, 3D culture showed positive cells at the periphery of the clumps and mostly negative cells in the centre (Figure 7(a)), whereas monolayer cells showed a uniform pattern of staining (Figure 7(b)). For the cell-surface glycoprotein CD44, the 3D culture was characterized by upregulation of CD44. There was positive staining of more than 50% of the cells, and dynamic changes were evident among the cells in each cell clump that represent cellular functional changes (Figure 7(c)); in contrast, less than 30% of monolayer cells were CD44 positive (Figure 7(d)). CA IX is a membrane protein related to hypoxia and a regulator of tissue pH. Cells in 3D culture showed enhanced expression represented by very strong membrane staining (Figure 7(e)); in contrast, cells in 2D culture showed very low positivity (Figure 7(f)). 

## 4. Discussion

Although there have been numerous investigations aimed at developing 3D cancer models, challenges still remain for initial attachment on porous scaffolds. In this study, we used the high-density random seeding technique to enhance the initial attachment of a non-small-cell lung cancer cell line. Our quantitative tests revealed that an attachment rate of 30–40% can be achieved. This represents an improvement over previous rates and allows controlled cell loading suitable for further evaluation with different surface coating and pore size. 

Our study of this 3D cancer model has demonstrated that the Variotis Tissue Scaffold is suitable for assessing cell adhesion, polarity, and morphology. The stiffness of Variotis Tissue Scaffolds is similar to that of biological connective tissue thus the NCI-H460 cells could readily attach and migrate along the scaffold surface. Mechanical forces have been demonstrated to alter gene expression and regulation of cell signaling, providing appropriate substrate stiffness vital to living cell functions. Recent studies have found that the rigidity of the matrix can influence stem cell differentiation, the migration of cells through cell membrane receptors, and activation of actin cytoskeleton [48–53]. Our results revealed that NCI-H460 cells grown on scaffolds formed tumor clusters of various sizes. The morphology of the cells appeared to represent their tissue origin, with distinct cellular membrane connection and distribution of necrotic cells. The porous nature of the scaffold allowed oxygen and nutrient to circulate freely in the culture. It also permitted the removal of detached cell and cell debris. In comparison, these specific growth patterns were not seen in the 2D monolayer culture which showed the typical pattern of cellular uniformity in size and shape and contact inhibition at confluence. 

The growth rate in 3D culture was very different from that in 2D culture. 3D culture maintained a slower proliferation rate with longer doubling time. The cells formed clusters between single layers of cells along the surface of scaffold to form multilayer clumps of sizes up to 500 *μ*m. Both H&E and Ki67 staining revealed the resting and nonviable cells in the center of cell clumps. This indicated that passive diffusion could no longer provide either sufficient nutrients or removal of waste products in the centre of the clumps. Lack of sufficient detoxification of this static system resulted in growth arrest and development of central necrosis. This pattern of central necrosis mimics tumor mass *in vivo* and provides a more realistic *in vitro* cancer model.

Considerable research has been devoted to determining how the surface of living cells senses their physical environment and translates into a biological response of altered protein and gene expression [6, 54–58]. CD44 is a membrane receptor for hyaluronic acid; it interacts with other cell-adhesion ligands such as collagens and matrix metalloproteinases. NCI-H460 cells had increased expression of CD44 in 3D scaffold compared with monolayer culture, indicating extracellular matrix protein activation. Furthermore, CD44 as a cell surface marker has been identified in some breast and prostate cancer stem cells. Although the pathways of the stem cell differentiation remain unknown, the expression of CD44 in our 3D culture suggests that the complex structure of scaffolds and the close communication of neighboring cells encouraged the differentiation of cancer stem cells.

It has been shown that CA IX regulates endothelial cell proliferation, and expression of CA IX has correlated with poor prognosis in various tumors [59, 60]. As cells form a 3D structure, they rely on their microenvironment to maintain the ability to proliferate and communicate. In our 3D culture, there was necrosis in the centre of the cell cluster and detachment of cells from the scaffold. This mimics necrosis seen clinically in the centre of a tumor mass. The overexpression of CA IX reflects these functional changes and demonstrates exchanges between cancer cells and their environment. 

Unlike the culture of cancer cells in matrix gel or cancer spheroid, our tumor 3D scaffold model proved very easy to handle. In addition to its use in immunohistochemistry this porous scaffold also provides a unique opportunity for studying soluble factors present in culture. For example, the hollow channel between the cell clusters allows soluble chemicals and secreted proteins to diffuse. Introducing specific culture conditions such as additional growth factors and chemokines will provide new insight into the mechanisms that regulate tumor proliferation and metastasis. The released soluble cellular products from scaffold culture can also be collected and detected by ELISA assays.

## 5. Conclusion

In conclusion, the present work compared the attachment and growth patterns of a non-small-cell cancer cell line in monolayer with a 3D culture in scaffold. Our experiments confirmed that the initial attachment to scaffold is enhanced by high-density seeding, and the behavior of cancer cell in our 3D model is similar to tumor growth *in vivo*. This model will provide the basis for future study using 3D cancer culture.

## List of Abbreviations

3D: 3 dimensional.
